# Supplementary material for: The five homologous CiaR-controlled Ccn sRNAs of Streptococcus pneumoniae modulate Zn-resistance
Source: bioRxiv. 2024 Apr 4:2023.11.07.565944. Originally published 2023 Nov 8. Preprint. [Version 2] doi: 10.1101/2023.11.07.565944 (PMC10659304; doi:10.1101/2023.11.07.565944)
Supplement: Supplement 1 [file media-1.pdf]

SUPPLEMENTAL MATERIAL

The five homologous CiaR-controlled Ccn sRNAs of *Streptococcus pneumoniae* modulate Zn-resistance.

Nicholas R. De Lay[1,4#], Nidhi Verma[1], Dhriti Sinha[1], Abigail Garrett[2], Maximillian K. Osterberg[3], Spencer Reiling[1], Daisy Porter[1], David P. Giedroc[3], and Malcolm E. Winkler[2]

[1]Department of Microbiology and Molecular Genetics, McGovern Medical School, University of Texas Health Science Center, Houston, TX 77030, USA

[2]Department of Biology, Indiana University Bloomington, Bloomington, Indiana 47405

[3]Department of Chemistry, Indiana University, Bloomington, Bloomington, Indiana 47405

[4]MD Anderson Cancer Center UTHealth Graduate School of Biomedical Sciences, University of Texas Health Science Center, Houston, TX 77030, USA

Running Title: Loss of the Ccn sRNAs cause Zn hypersensitivity

Keywords: small RNA; post-transcriptional regulation; Zn homeostasis; Mn homeostasis; CiaRH

[#] Correspondence should be sent to Nicholas R. De Lay (Tel: +1 (713) 500-6293; Email: nicholas.r.delay@uth.tmc.edu)

TABLE OF CONTENTS

SUPPLEMENTAL TABLES

TABLE S1. *S. pneumoniae* strains used in this study

TABLE S2. Primers used to construct mutants used in this study

TABLE S3. Comparison of gene expression *S. pneumoniae* D39 and derived Δ*ccnABCDE* strains in BHI broth by RNA-seq. (see Excel spreadsheet)

TABLE S4. Comparison of gene expression *S. pneumoniae* TIGR4 and derived Δ*ccnABCDE* strains in BHI broth by RNA-seq. (see Excel spreadsheet)

TABLE S5. Comparison of gene expression *S. pneumoniae* D39 and derived Δ*ccnABCDE* strains in BHI broth with 0.2 mM $ZnSO_4$ by RNA-seq. (see Excel spreadsheet)

TABLE S6. Oligonucleotide primers used for qRT-PCR

SUPPLEMENTAL FIGURES

FIGURE S1. Growth phenotypes of *S. pneumoniae* D39 derived strains harboring deletion of individual *ccn* genes.

FIGURE S2. Virulence phenotypes of *S. pneumoniae* strains harboring deletion of individual *ccn* genes.

FIGURE S3. Growth phenotypes of *S. pneumoniae* TIGR4 derived strains harboring deletion of the *ccn* genes.

FIGURE S4. Doubling times of *S. pneumoniae* D39, Δ*ccnABCDE* mutant, or Δ*ccnABCDE* mutant strain complemented with *ccnA, ccnB,* and *ccnC* in C medium alone or supplemented with Zn.

FIGURE S5. Growth phenotypes of *S. pneumoniae* D39 and derived strains harboring deletion of the *ccn* genes and/or *psaR* or *mntE*.

SUPPLEMENTAL REFERENCES

SUPPLEMENTAL TABLES

Table S1. *S. pneumoniae* strains used in this study

Strain	Genotype (description)[a]	Antibiotic resistance[b]	Reference or source
K272	D39 Δ*cps* Δ*pnp*::P_c-[*kan rpsL*$^+$]	Kan^R	(*1*)
IU1781	D39 *rpsL1*	Str^R	(*2*)
IU5122	D39 Δ*cps rpsL1* CEP::P_c-[*kan rpsL*$^+$]	Kan^R	(*3*)
IU5382	D39 Δ*cps* CEP::P_{fcsK}-*ftsE*	None	(*3*)
IU9681	D39 Δ*cps hlpA hlpA-mkate-cat*	Cm^R	Malcolm Winkler
IU10508	D39 Δ*cps* Δ*bgaA*::*kan*-T_1-T_2-P_{spd_1874}-*lacZ*	Kan^R	(*4*)
IU11966	TIGR4	None	(*5, 6*)
IU12001	TIGR4 Δ*cps*	None	(*7*)
NRD10068	D39 *rpsL1* Δ*ccnA*::P_c-[*kan rpsL*$^+$]	Kan^R	This study
NRD10069	D39 *rpsL1* Δ*ccnB*::P_c-[*kan rpsL*$^+$]	Kan^R	This study
NRD10070	D39 *rpsL1* Δ*ccnC*::P_c-[*kan rpsL*$^+$]	Kan^R	This study
NRD10071	D39 *rpsL1* Δ*ccnD*::P_c-[*kan rpsL*$^+$]	Kan^R	This study
NRD10072	D39 *rpsL1* Δ*ccnE*::P_c-[*kan rpsL*$^+$]	Kan^R	This study
NRD10073	D39 *rpsL1* Δ*ccnA*	Str^R	This study
NRD10074	D39 *rpsL1* Δ*ccnB*	Str^R	This study
NRD10075	D39 *rpsL1* Δ*ccnC*	Str^R	This study
NRD10076	D39 *rpsL1* Δ*ccnD*	Str^R	This study
NRD10077	D39 *rpsL1* Δ*ccnE*	Str^R	This study
NRD10078	D39 *rpsL1* Δ*ccnB* Δ*ccnC*::P_c-[*kan rpsL*$^+$]	Kan^R	This study
NRD10079	D39 *rpsL1* Δ*ccnB* Δ*ccnC*	Str^R	This study
NRD10080	D39 *rpsL1* Δ*ccnB* Δ*ccnC* Δ*ccnD*::P_c-[*kan rpsL*$^+$]	Kan^R	This study
NRD10081	D39 *rpsL1* Δ*ccnB* Δ*ccnC* Δ*ccnD*	Str^R	This study
NRD10083	D39 *rpsL1* Δ*ccnB* Δ*ccnC* Δ*ccnD* Δ*ccnE*::P_c-[*kan rpsL*$^+$]	Kan^R	This study
NRD10085	D39 *rpsL1* Δ*ccnB* Δ*ccnC* Δ*ccnD* Δ*ccnE*	Str^R	This study
NRD10171	D39 *rpsL1* Δ*ccnAB*::P_c-[*kan rpsL*$^+$] Δ*ccnC* Δ*ccnD* Δ*ccnE*	Kan^R	This study
NRD10176	D39 *rpsL1* Δ*ccnAB* Δ*ccnC* Δ*ccnD* Δ*ccnE*	Str^R	This study
NRD10220	TIGR4 *rpsL1*	Str^R	This study
NRD10225	TIGR4 *rpsL1* Δ*ccnA*::P_c-[*kan rpsL*$^+$]	Kan^R	This study
NRD10230	TIGR4 *rpsL1* Δ*ccnA*	Str^R	This study
NRD10247	TIGR4 *rpsL1* Δ*ccnA* Δ*ccnB*::P_c-[*kan rpsL*$^+$]	Kan^R	This study
NRD10249	TIGR4 *rpsL1* Δ*ccnA* Δ*ccnB*	Str^R	This study
NRD10251	TIGR4 *rpsL1* Δ*ccnA* Δ*ccnB* Δ*ccnC*::P_c-[*kan rpsL*$^+$]	Kan^R	This study
NRD10254	TIGR4 *rpsL1* Δ*ccnA* Δ*ccnB* Δ*ccnC*	Str^R	This study
NRD10257	TIGR4 *rpsL1* Δ*ccnA* Δ*ccnB* Δ*ccnC* Δ*ccnD*::P_c-[*kan rpsL*$^+$]	Kan^R	This study
NRD10261	TIGR4 *rpsL1* Δ*ccnA* Δ*ccnB* Δ*ccnC* Δ*ccnD*	Str^R	This study
NRD10265	TIGR4 *rpsL1* Δ*ccnA* Δ*ccnB* Δ*ccnC* Δ*ccnD* Δ*ccnE*::P_c-[*kan rpsL*$^+$]	Kan^R	This study
NRD10266	TIGR4 *rpsL1* Δ*ccnA* Δ*ccnB* Δ*ccnC* Δ*ccnD* Δ*ccnE*	Str^R	This study

NRD10306	TIGR4 *rpsLK56T* Δ*ccnA*::P$_c$-[*kan rpsL*$^+$]	Kan^R	This study
NRD10311	TIGR4 *rpsL*$^+$-*rpsG*$^+$-*cat*	Cm^R	This study
NRD10312	TIGR4 *rpsLK56T* Δ*ccnA*	Str^R	This study
NRD10322	TIGR4 *rpsLK56T* Δ*ccnA* Δ*ccnB*::P$_c$-[*kan rpsL*$^+$]	Kan^R	This study
NRD10324	TIGR4 *rpsLK56T* Δ*ccnA* Δ*ccnB*	Str^R	This study
NRD10330	TIGR4 *rpsLK56T* Δ*ccnA* Δ*ccnB* Δ*ccnC*::P$_c$-[*kan rpsL*$^+$]	Kan^R	This study
NRD10332	TIGR4 *rpsLK56T* Δ*ccnA* Δ*ccnB* Δ*ccnC*	Str^R	This study
NRD10336	TIGR4 *rpsLK56T* Δ*ccnA* Δ*ccnB* Δ*ccnC* Δ*ccnD*::P$_c$-[*kan rpsL*$^+$]	Kan^R	This study
NRD10340	TIGR4 *rpsLK56T* Δ*ccnA* Δ*ccnB* Δ*ccnC* Δ*ccnD*	Str^R	This study
NRD10344	TIGR4 *rpsLK56T* Δ*ccnA* Δ*ccnB* Δ*ccnC* Δ*ccnD* Δ*ccnE*::P$_c$-[*kan rpsL*$^+$]	Kan^R	This study
NRD10345	TIGR4 *rpsLK56T* Δ*ccnA* Δ*ccnB* Δ*ccnC* Δ*ccnD* Δ*ccnE*	Str^R	This study
NRD10346	TIGR4 *rpsL*$^+$-*rpsG*$^+$-*cat* Δ*ccnA* Δ*ccnB* Δ*ccnC* Δ*ccnD* Δ*ccnE*	Cm^R	This study
NRD10390	D39 *rpsL1* CEP::P$_c$-[*kan rpsL*$^+$]		This study
NRD10391	D39 *rpsL1* Δ*ccnAB* Δ*ccnC* Δ*ccnD* Δ*ccnE* CEP::P$_c$-[*kan rpsL*$^+$]	Kan^R	This study
NRD10393	D39 *rpsL1* Δ*ccnAB* Δ*ccnC* Δ*ccnD* Δ*ccnE* CEP::T$_1$-T$_2$-*ccnA*-*ccnB*	Str^R	This study
NRD10394	D39 *rpsL1* Δ*ccnAB* Δ*ccnC* Δ*ccnD* Δ*ccnE* CEP::T$_1$-T$_2$-*ccnC*	Str^R	This study
NRD10396	D39 *rpsL1* Δ*ccnAB* Δ*ccnC* Δ*ccnD* Δ*ccnE* CEP::T$_1$-T$_2$-*ccnA*-*ccnB* Δ*bgaA*::*kan*-*T$_1$*-*T$_2$*-*ccnD*	Str^R, Kan^R	This study
NRD10397	D39 *rpsL1* Δ*ccnAB* Δ*ccnC* Δ*ccnD* Δ*ccnE* CEP::T$_1$-T$_2$-*ccnC* Δ*bgaA*::*kan*-*T$_1$*-*T$_2$*-*ccnD*	Str^R, Kan^R	This study
NRD10441	D39 *rpsL1* Δ*psaR*::P$_c$-[*kan rpsL*$^+$]	Kan^R	This study
NRD10442	D39 *rpsL1* Δ*mntE*::P$_c$-[*kan rpsL*$^+$]	Kan^R	This study
NRD10443	D39 *rpsL1* Δ*ccnAB* Δ*ccnC* Δ*ccnD* Δ*ccnE* Δ*psaR*::P$_c$-[*kan rpsL*$^+$]	Kan^R	This study
NRD10444	D39 *rpsL1* Δ*ccnAB* Δ*ccnC* Δ*ccnD* Δ*ccnE* Δ*mntE*::P$_c$-[*kan rpsL*$^+$]	Kan^R	This study
NRD10447	D39 *rpsL1* Δ*psaR*	Str^R	This study
NRD10448	D39 *rpsL1* Δ*mntE*	Str^R	This study
NRD10449	D39 *rpsL1* Δ*ccnAB* Δ*ccnC* Δ*ccnD* Δ*ccnE* Δ*psaR*	Str^R	This study
NRD10450	D39 *rpsL1* Δ*ccnAB* Δ*ccnC* Δ*ccnD* Δ*ccnE* Δ*mntE*	Str^R	This study
NRD10533	D39 *rpsL1* Δ*sodA::erm*	Erm^R	This study
NRD10534	D39 *rpsL1* Δ*ccnAB* Δ*ccnC* Δ*ccnD* Δ*ccnE* Δ*sodA::erm*	Erm^R	This study
TIGR4S	TIGR4 *rpsLK56T*	Str^R	This study
TIGR4SΔcps	TIGR4 *rpsLK56T* Δ*cps*	Str^R	This study

[a] Strains were constructed by transformation of amplicons into the indicated recipient strain as described in Materials and Methods. Primers used to synthesize fusion amplicons are listed in Table S2.

[b]Antibiotic resistance markers: Kan, kanamycin; Str, streptomycin; Erm, Erythromycin.

Table S2. Primers used to construct mutants used in this study

Primer name	Primer Sequence (5'-3')	Template	Product
**For construction of strain NRD10068 (Δ*ccnA::*P_c-[kanR-rpsL$^+$])**			
Spn001	TCATAGACAAGGCGACTGGTAAGG	IU1945	Upstream of *ccnA*
Spn002	CCATTAAAAATCAAACGGATCCTAAAAAAAGTTTAGGATTTTATTAAATAAAGTTAGG		
kanrpsL For	TAGGATCCGTTTGATTTTTAATGGATAATG	K272	P_c-[*kan rpsL*$^+$][b]
kanrpsL rev	GGGCCCCTTTCCTTATGCTTTTG		
Spn003	TCCAAAAGCATAAGGAAAGGGGCCCGGCTTTTTGCGTGGTGAGGTGCTGGTG	IU1945	Downstream of *ccnA*
Spn004	GGCCCAAATAAGAGCACATGATCC		
**For construction of strain NRD10069 (Δ*ccnB::*P_c-[kanR-rpsL$^+$])**			
Spn005	ATCATAGACAAGGCGACTGGTAAGGC	IU1945	Upstream of *ccnB*
Spn006	CCATTAAAAATCAAACGGATCCTAGGAGGTCTTTATTTAATAACTACATG		
kanrpsL For	TAGGATCCGTTTGATTTTTAATGGATAATG	K272	P_c-[*kan rpsL*$^+$][b]
kanrpsL rev	GGGCCCCTTTCCTTATGCTTTTG		
Spn007	TCCAAAAGCATAAGGAAAGGGGCCCCAGGTGGAGTTTTTTAGCTCTATTTCAG	IU1945	Downstream of *ccnB*
Spn008	CCAAATGAACACTACGACTACCCTCACC		
**For construction of strains NRD10070 and NRD10078 (Δ*ccnC::*P_c-[kanR-rpsL$^+$])**			
Spn036	GCCTTATCATATCGAGTTGGATCGCTTGC	IU1945	Upstream of *ccnC*
Spn034	CACATTATCCATTAAAAATCAAACGGATCCTAGTCTATAGTATACCCGACCTATCTTAAAC		
kanrpsL For	TAGGATCCGTTTGATTTTTAATGGATAATG	K272	P_c-[*kan rpsL*$^+$][b]
kanrpsL rev	GGGCCCCTTTCCTTATGCTTTTG		
Spn035	CGTCCAAAAGCATAAGGAAAGGGGCCCGTGTTGGGATTCATGATATAATAATAAAATCG	IU1945	Downstream of *ccnC*
Spn037	TAATCCCCATCAATGACCCCAACTGAGT		
**For construction of strains NRD10071 and NRD10080 (Δ*ccnD::*P_c-[kanR-rpsL$^+$])**			
**Spn020**	AATGAGTTAGAGCCTGGGGATGTCC	IU1945	Upstream of *ccnD*
**Spn018**	ATCCATTAAAAATCAAACGGATCCTAGAACTTAGTGTACACTCCCTAGCTTAAAGTTTCC		
kanrpsL For	TAGGATCCGTTTGATTTTTAATGGATAATG	K272	P_c-[*kan rpsL*$^+$][b]

kanrpsL rev	GGGCCCCTTTCCTTATGCTTTTG		
Spn019	CGTCCAAAAGCATAAGGAAAGGGG CCCGAAAAATGGGCTTGGTGCCTGA GAAT	IU1945	Downstream of *ccnD*
Spn021	GTCAGGTAATTCTCCAAGGGAATGG		
**For construction of strains NRD10072 and NRD10083 (Δ*ccnE::*P_c-[kanR-rpsL$^+$])**			
Spn023	GTATCGGTGACACCTATTTCTCTGA	IU1945	Upstream of *ccnE*
Spn022	CACATTATCCATTAAAAATCAAACGG ATCCTAGATTATAGTATACACATCTA ATCTT		
kanrpsL For	TAGGATCCGTTTGATTTTTAATGGAT AATG	K272	P_c-[*kan rpsL*$^+$][b]
kanrpsL rev	GGGCCCCTTTCCTTATGCTTTTG		
Spn024	CGTCCAAAAGCATAAGGAAAGGGG CCCTCATTCATGATATAATAGAAGCA AACGGAG	IU1945	Downstream of *ccnE*
Spn025	CACTGATTTCAGAACTCTCTACTTCT ATCC		
**For construction of strain NRD10073 (Δ*ccnA*)**			
Spn013	CAGTGGAAAAGCATGCGGAGGATTT G	IU1945	Upstream of *ccnA*
Spn015	TCTATCACCAGCACCTCACCACGCG ACTATATAATACTAGACCATCCT		
Spn014	AGGATGGTCTAGTATTATATAGTCG CGTGGTGAGGTGCTGGTGATAGA	IU1945	Downstream of *ccnA*
Spn004	GGCCCAAATAAGAGCACATGATCC		
**For construction of strain NRD10074 (Δ*ccnB*)**			
Spn013	CAGTGGAAAAGCATGCGGAGGATTT G	IU1945	Upstream of *ccnB*
Spn028	GTCCCCAAAAGCCTGAAATAGAGCT AACTACATGATACAAGACGAAACTTA AAAC		
Spn029	AAGTTTCGTCTTGTATCATGTAGTTA GCTCTATTTCAGGCTTTTGGGGACT ATTC	IU1945	Downstream of *ccnB*
Spn004	GGCCCAAATAAGAGCACATGATCC		
**For construction of strains NRD10075 and NRD10079 (Δ*ccnC*)**			
Spn036	GCCTTATCATATCGAGTTGGATCGC TTGC	IU1945	Upstream of *ccnC*
5-ccnCcleanKO Rev	CGATTTTATTATTATATCATGAATCC CAACACGTCTATAGTATACCCGACC TATCTTAAAC		
3'ccnCcleanKO For	GTTTAAGATAGGTCGGGTATACTAT AGACGTGTTGGGATTCATGATATAA TAATAAAATCG	IU1945	Downstream of *ccnC*
Spn037	TAATCCCCATCAATGACCCCAACTG AGT		
**For construction of strains NRD10076 and NRD10081 (Δ*ccnD*)**			
Spn020	AATGAGTTAGAGCCTGGGGATGTCC	IU1945	

Spn031	TTCTCAGGCACCAAGCCCATTTTTCGAACTTAGTGTACACTCCCTAGCTT		Upstream of *ccnD*
Spn030	CTTTAAGCTAGGGAGTGTACACTAAGTTCGAAAAATGGGCTTGGTGCCTGAGAAT	IU1945	Downstream of *ccnD*
Spn021	GTCAGGTAATTCTCCAAGGGAATGG		
**For construction of strains NRD10077 and NRD10085 (Δ*ccnE*)**			
Spn023	GTATCGGTGACACCTATTTCTCTGA	IU1945	Upstream of *ccnE*
Spn033	CCGTCCTCCGTTTGCTTCTATTATATCATGAATGAGATTATAGTATACACATCTAATCTT		
Spn032	AAGATTAGATGTGTATACTATAATCTCATTCATGATATAATAGAAGCAAACGGAGGACGG	IU1945	Downstream of *ccnE*
Spn025	CACTGATTTCAGAACTCTCTACTTCTATCC		
**For construction of strain NRD10171 (Δ*ccnAB::*P_c-[kanR-rpsL$^+$])**			
Spn001	TCATAGACAAGGCGACTGGTAAGG	IU1945	Upstream of *ccnA*
Spn002	CCATTAAAAATCAAACGGATCCTAAAAAAAGTTTAGGATTTTATTAAATAAAGTTAGG		
kanrpsL For	TAGGATCCGTTTGATTTTTAATGGATAATG	K272	P_c-[*kan rpsL*$^+$][b]
kanrpsL rev	GGGCCCCTTTCCTTATGCTTTTG		
Spn007	TCCAAAAGCATAAGGAAAGGGGCCCCAGGTGGAGTTTTTTAGCTCTATTTCAG	IU1945	Downstream of *ccnB*
Spn008	CCAAATGAACACTACGACTACCCTCACC		
**For construction of strain NRD10176 (Δ*ccnAB*)**			
Spn001	TCATAGACAAGGCGACTGGTAAGG	IU1945	Upstream of *ccnA*
53-ccnAB For2	GCAAAATTTAAGGATGGTCTAGTATTATATAGTCAGCTCTATTTCAGGCTTTTGGG		
53-ccnAB Rev2	CCCAAAAGCCTGAAATAGAGCTGACTATATAATACTAGACCATCCTTAAATTTTGC	IU1945	Downstream of *ccnB*
Spn008	CCAAATGAACACTACGACTACCCTCACC		
**For construction of strains NRD10225 and NRD10306 (Δ*ccnA::*P_c-[kanR-rpsL$^+$])**			
T4ccnA For	CAGTGGAAAAGCATGCGGAGG	TIGR4	Upstream of *ccnA*
5-T4ccnA-rpsL Rev	CATTATCCATTAAAAATCAAACGGATCCTAGACTATATGATACTAGACCATCCTTAAAC		
kanrpsL For	TAGGATCCGTTTGATTTTTAATGGATAATG	K272	P_c-[*kan rpsL*$^+$][b]
kanrpsL rev	GGGCCCCTTTCCTTATGCTTTTG		
3-T4ccnA-rpsL For	CAAAAGCATAAGGAAAGGGGCCCGGTACGACGGGCATGTCG	TIGR4	Downstream of *ccnA*

T4ccnA Rev	GGCTCATGACTTGGACAATGG		
**For construction of strains NRD10230 and NRD10312 (Δ*ccnA*)**			
T4ccnA For	CAGTGGAAAAGCATGCGGAGG	TIGR4	Upstream of *ccnA*
5-T4ccnA-cln Rev	CGACATGCCCGTCGTACCGCAGACT ATATGATACTAGACCATCCTTAAAC		
3-T4ccnA-cln-For	GTTTAAGGATGGTCTAGTATCATATA GTCTGCGGTACGACGGCATGTCG	TIGR4	Downstream of *ccnA*
T4ccnA Rev	GGCTCATGACTTGGACAATGG		
**For construction of strain NRD10247 (Δ*ccnB::*P_c-[kanR-rpsL$^+$])**			
T4ccnB For	GCCATTGTCCAAGTCATGAGC	TIGR4	Upstream of *ccnB*
5-T4ccnB-rpsL Rev	CATTATCCATTAAAAATCAAACGGAT CCTACTACATGATACAAGACGAAAC TTAAAACTAGC		
kanrpsL For	TAGGATCCGTTTGATTTTTAATGGAT AATG	K272	P_c-[*kan rpsL*$^+$][b]
kanrpsL rev	GGGCCCCTTTCCTTATGCTTTTG		
3-T4ccnB-rpsL For	CAAAAGCATAAGGAAAGGGGCCCA GCTCTATTTCAGGATTTTTGGGAC	TIGR4	Downstream of *ccnB*
T4ccnB Rev	GAGGACATCTCCAGGCTCTAAATC		
**For construction of strains NRD10249 and NRD10322 (Δ*ccnB*)**			
T4ccnB For	GCCATTGTCCAAGTCATGAGC	TIGR4	Upstream of *ccnB*
5-T4ccnB-cln Rev	GTCCCAAAAATCCTGAAATAGAGCT AACTACATGATACAAGACGAAACTTA AAACTAGC		
3-T4ccnB-cln-For	GCTAGTTTTAAGTTTCGTCTTGTATC ATGTAGTTAGCTCTATTTCAGGATTT TTGGGAC	TIGR4	Downstream of *ccnB*
T4ccnB Rev	GAGGACATCTCCAGGCTCTAAATC		
**For construction of strains NRD10251 and NRD10330 (Δ*ccnC::*P_c-[kanR-rpsL$^+$])**			
T4ccnC For	GCCTTATCATATCGAGTTGGATCGC TTGC	TIGR4	Upstream of *ccnC*
5-T4ccnC-rpsL Rev	CATTATCCATTAAAAATCAAACGGAT CCTAGTCTATAGTATACCCGACCTAT CTTAAAC		
kanrpsL For	TAGGATCCGTTTGATTTTTAATGGAT AATG	K272	P_c-[*kan rpsL*$^+$][b]
kanrpsL rev	GGGCCCCTTTCCTTATGCTTTTG		
3-cT4cnC-rpsL For	CAAAAGCATAAGGAAAGGGGCCCG TGTTGGGATTCATGATATAATAATAA AATCG	TIGR4	Downstream of *ccnC*
T4ccnC Rev	CTTCAAGAACAACCTCTTGGTCTG		
**For construction of strains NRD10254 and NRD10332 (Δ*ccnC*)**			
T4ccnC For	GCCTTATCATATCGAGTTGGATCGC TTGC	TIGR4	Upstream of *ccnC*
5-T4ccnC-cln Rev	CGATTTTATTATTATATCATGAATCC CAACACGTCTATAGTATACCCGACC TATCTTAAAC		

3-T4ccnC-cln-For	GTTTAAGATAGGTCGGGTATACTATAGACGTGTTGGGATTCATGATATAATAATAAAATCG	TIGR4	Downstream of *ccnC*
T4ccnC Rev	CTTCAAGAACAACCTCTTGGTCTG		
**For construction of strains NRD10257 and NRD10336 (Δ*ccnD::*P_c-[kanR-rpsL$^+$])**			
T4ccnD For	AATGATTTAGAGCCTGGAGATGTCC	TIGR4	Upstream of *ccnD*
5-T4ccnD-rpsL Rev	CATTATCCATTAAAAATCAAACGGATCCTAGAACTTAGTGTACACTCCCTAGCTTAAAG		
kanrpsL For	TAGGATCCGTTTGATTTTTAATGGATAATG	K272	P_c-[*kan rpsL*$^+$][b]
kanrpsL rev	GGGCCCCTTTCCTTATGCTTTTG		
3-T4ccnD-rpsL For	CAAAAGCATAAGGAAAGGGGCCCGAAAAATGGGCTTGGTGCCTG	TIGR4	Downstream of *ccnD*
T4ccnD Rev	GTCAGGTAATTCTCCAAGGGAATGG		
**For construction of strains NRD10261 and NRD10340 (Δ*ccnD*)**			
T4ccnD For	AATGATTTAGAGCCTGGAGATGTCC	TIGR4	Upstream of *ccnD*
5-T4ccnD-cln Rev	CAGGCACCAAGCCCATTTTTCGAACTTAGTGTACACTCCCTAGCTTAAAG		
3-T4ccnD-cln-For	CTTTAAGCTAGGGAGTGTACACTAAGTTCGAAAAATGGGCTTGGTGCCTG	TIGR4	Downstream of *ccnD*
T4ccnD Rev	GTCAGGTAATTCTCCAAGGGAATGG		
**For construction of strains NRD10265 and NRD10344 (Δ*ccnE::*P_c-[kanR-rpsL$^+$])**			
T4ccnE For	GTATCGGTGACACCTATTTCTCTGA	TIGR4	Upstream of *ccnE*
5-T4ccnE-rpsL Rev	CATTATCCATTAAAAATCAAACGGATCCTAGATTATAGTATACACGTCTAATCTTAAATAGAAC		
kanrpsL For	TAGGATCCGTTTGATTTTTAATGGATAATG	K272	P_c-[*kan rpsL*$^+$][b]
kanrpsL rev	GGGCCCCTTTCCTTATGCTTTTG		
3-T4ccnE-rpsL For	CAAAAGCATAAGGAAAGGGGCCCTCATTCATGATATAATAGAAGCAAACGG	TIGR4	Downstream of *ccnE*
T4ccnE Rev	GCTATCTGGTATCATTTCTGGAGCC		
**For construction of strains NRD10266 and NRD10345 (Δ*ccnE*)**			
T4ccnE For	GTATCGGTGACACCTATTTCTCTGA	TIGR4	Upstream of *ccnE*
5-T4ccnE-cln Rev	CCGTTTGCTTCTATTATATCATGAATGAGATTATAGTATACACGTCTAATCTTAAATAGAAC		
3-T4ccnE-cln-For	GTTCTATTTAAGATTAGACGTGTATACTATAATCTCATTCATGATATAATAGAAGCAAACGG	TIGR4	Downstream of *ccnE*
T4ccnE Rev	GCTATCTGGTATCATTTCTGGAGCC		
**For construction of strains NRD10311 and NRD10346 (*rpsL*$^+$*-rpsG*$^+$*-cat*)**			
KK265	GTGAATTGGTCGGAATTGTAGCTAACAG	TIGR4	Upstream of *rpsL* + stop codon of *rpsG*
KK529	GCCTCCTAAATTTACCAACGGAAGTGTGCGAATG		

KK530	CCGTTGGTAAATTTAGGAGGCATAT CAAATGAACT	IU9681	*cat* cassette
KK531	CGCATCCTATCTTATAAAAGCCAGT CATTAGGCC		
KK532	CTGGCTTTTATAAGATAGGATGCGA AAGCGTTAAG	TIGR4	Stop codon of *rpsG* + downstream of *rpsG*
KK270	CAAGGATATCCGTACCAAGGTCGTT AG		
**For construction of strains NRD10390 and NRD10391 (*CEP::*P_c-[kanR-rpsL$^+$])**			
AmiF For	CCAGCTGTGATTGCAAATCTCCATG	IU5122	Upstream of *amiF* + downstream of *amiF*
TreR Rev	GGCTTCTTGTTCAAATTTTCCCATTT GATTCTC		
**For construction of strain NRD10393 (*CEP::*T_1-T_2-*ccnA-ccnB*)**			
5-AmiF-Ptet for	CTGGCTGACTAGGAGGAAGG	IU5382	Upstream of *amiF* + T_1-T_2 terminator
5-ccnABCEP_rev	CCAAGTCATCCCTATACAATTATAGG TGGGTAGAAACGCAAAAAGGCCATC		
M-ccnAB For	GATGGCCTTTTTGCGTTTCTACCCA CCTATAATTGTATAGGGATGACTTG G	IU1945	101 nt upstream of *ccnA* + 139 nt downstream of *ccnB*
M-ccnAB Rev	GCTCCCTTTTTTAATGGTAACACCG CTGAAGCGACCACAGACC		
3-ccnABCEP_ for	AGGTCTGTGGTCGCTTCAGCGGTGT TACCATTAAAAAAGGGAGC	IU5382	26 nt of spd_1082 + downstream of *amiF*
3-TreR-Ptet rev	CCCATTTGATTCTCCTTATACTTGTC AAAGC		
**For construction of strain NRD10394 (*CEP::*T_1-T_2-*ccnC*)**			
5-AmiF-Ptet for	CTGGCTGACTAGGAGGAAGG	IU5382	Upstream of *amiF* + T_1-T_2 terminator
5-ccnCCEP_rev	CGAACGCCAACCAATTCACTCGTAG AAACGCAAAAAGGCCATC		
M-ccnC For	GATGGCCTTTTTGCGTTTCTACGAG TGAATTGGTTGGCGTTCG	IU1945	133 nt upstream of *ccnC* + 111 nt downstream of *ccnC*
M-ccnC Rev	GCTCCCTTTTTTAATGGTAACACCG CCTCTCTCAAAGCCTCCC		
3-ccnCCEP_ for	GGGAGGCTTTGAGAGAGGCGGTGT TACCATTAAAAAAGGGAGC	IU5382	26 nt of spd_1082 + downstream of *amiF*
3-TreR-Ptet rev	CCCATTTGATTCTCCTTATACTTGTC AAAGC		
**For construction of strains NRD10396 and NRD10397 (Δ*bgaA-kan*-T_1-T_2-*ccnD*)**			
5-BgaFor2	CTGGTGATATCAAAGCAATCCTTGG	IU10508	Upstream of bgaA + T_1-T_2 terminator
5-ccnDBga Rev	CCATTTCTCCTGCCAATTTTTCTTGG TAGAAACGCAAAAAGGCCATC		
M-ccnD For	GATGGCCTTTTTGCGTTTCTACCAA GAAAAATTGGCAGGAGAAATGG	IU1945	86 nt upstream of *ccnD* + 55 nt downstream of *ccnD*
M-ccnD Rev	GCAACTGGTTTATGAGAAAGTAAGT TCCCCCATTTTCTTCTATCACTAAGC		
3-ccnDBga For	GCTTAGTGATAGAAGAAAATGGGGG AACTTACTTTCTCATAAACCAGTTGC	IU10508	1501 nt of *bgaA* +

3-Bga-rpsLKan Rev	CTGGTTTTTCCTTAGTCAACTGGATACGG		downstream of *bgaA*
**For construction of strains NRD10441 and NRD10443 (Δ*psaR::*P_c-[kanR-rpsL^{+}])**			
5-psaR for	GAGGCTACCCTGCCTCTACTC	IU1945	Upstream of *psaR*
5-psaR rpsL rev	CATTATCCATTAAAAATCAAACGGATCCTATAGATAGTCTTCTTTGTTTGGGGTC		
kanrpsL For	TAGGATCCGTTTGATTTTTAATGGATAATG	K272	P_c-[*kan rpsL*$^{+}$][b]
kanrpsL rev	GGGCCCCTTTCCTTATGCTTTTG		
3-psaR rpsL for	CAAAAGCATAAGGAAAGGGGCCCATTGCAAAACAACTCTATGTCGAG	IU1945	Downstream of *psaR*
3-psaR rev	CCAGAGAGCAAGAGCCACTC		
**For construction of strains NRD10442 and NRD10444 (Δ*mntE::*P_c-[kanR-rpsL^{+}])**			
5-mntE for	CCGCATCTTGAAGCATACCAGC	IU1945	Upstream of *mntE*
5-mntE rpsL rev	CATTATCCATTAAAAATCAAACGGATCCTACTCAGCTAACTTGAGATTTGAGATAG		
kanrpsL For	TAGGATCCGTTTGATTTTTAATGGATAATG	K272	P_c-[*kan rpsL*$^{+}$][b]
kanrpsL rev	GGGCCCCTTTCCTTATGCTTTTG		
3-mntE rpsL for	CAAAAGCATAAGGAAAGGGGCCCTGGCAAAATATCTTTCATCAAGAAACC	IU1945	Downstream of *mntE*
3-mntE rev	GGAAGAAAGTCATCGAGTTTCAGG		
**For construction of strains NRD10447 and NRD10449 (Δ*psaR*)**			
5-psaR for	GAGGCTACCCTGCCTCTACTC	IU1945	Upstream of *psaR*
5-psaR cln rev	CTCGACATAGAGTTGTTTTGCAATTAGATAGTCTTCTTTGTTTGGGGTC		
3-psaR cln for	GACCCCAAACAAAGAAGACTATCTAATTGCAAAACAACTCTATGTCGAG	IU1945	Downstream of *psaR*
3-psaR rev	CCAGAGAGCAAGAGCCACTC		
**For construction of strains NRD10448 and NRD10450 (Δ*mntE*)**			
5-mntE for	CCGCATCTTGAAGCATACCAGC	IU1945	Upstream of *mntE*
5-mntE cln rev	GGTTTCTTGATGAAAGATATTTTGCCACTCAGCTAACTTGAGATTTGAGATAG		
3-mntE cln for	CTATCTCAAATCTCAAGTTAGCTGAGTGGCAAAATATCTTTCATCAAGAAACC	IU1945	Downstream of *mntE*
3-mntE rev	GGAAGAAAGTCATCGAGTTTCAGG		
**For construction of strains NRD10533 and NRD10534 (Δ*sodA::erm*)**			
5-sodA For	CAGGGTCAGTTTGAAGTGATGAAGAG	IU1945	Upstream of *sodA*
5-sodA-erm Rev	TATTTTATATTTTTGTTCATCTGTAATACCTCTTTTTCTTTCTATATG		
M-sodA-erm For	GAAAAAGAGGTATTACAGATGAACAAAAATATAAAATATTCTC	NRD10491[c]	erm[b]
M-sodA-erm Rev	CCTCCAACTATCATTATTTCCTCCCGTTAAATAATAGATAAC		

3-sodA-erm For	TAACGGGAGGAAATAATGATAGTTG GAGGGAAGAATTGTTC	IU1945	stop codon of *sodA* + downstream
3-sodA Rev	CCATAGTGTTGACGCATGAGTACAG		
**For construction of strains TIGR4S and TIGR4SΔcps (*rpsLK56T*)**			
HE01	GCCGTAGTCATCTTTCTTGGCATC	IU11966	Upstream *rpsL* + 181 nt of *rpsL* with A167C change
HE02	CTGAGTTAGGTTTTGTAGGTGTCATT GTTC		
HE03	GAACAATGACACCTACAAAACCTAA CTCAG	IU11966	263 nt of *rpsL* with A167C change + downstream
HE04	CTAATTTGAACCCGGGCTAAAGTTA G		

[a] Genomic DNA of indicated *S. pneumoniae* strains was used as templates for PCR reactions, except for P_c-[*kan-rpsL*$^+$] and P_c-*erm* cassettes.

[b] P_c-*erm* and P_c-[*kan-rpsL*$^+$] cassettes are described in (Tsui *et al.*, 2010) (*8*).

[c] NRD10491 is an unpublished strain with genotype D39 Δcps pnp-3XFLAG-P_c-*erm*.

Table S6. Oligonucleotide primers and probes used for qRT-PCR and northern blots.

**Primer**	**Primer or probe sequence (5'-3')**	**Gene**
D39_czcD_For	CGGGCTCTGTTCTAGTCATTT	*czcD*
D39_czcD_Rev	CCAGACTCGCTAACAGATTGAT	*czcD*
TIGR4_czcD_For	TGGTGGTTGGTAAGGGAAAG	*czcD*
TIGR4_czcD_Rev	AGAACAATCGCCATCAGGATAA	*czcD*
piuB_For	CGGCTCAGTCACAGAAGTTATC	*piuB*
piuB_Rev	GCCTAAGAAGAGCCACTCATAC	*piuB*
spd_1267_For	TGGATGAGCCTTCATCGAATTTA	*spd_1267*
spd_1267_Rev	CACGGTCAACTATGTCCATCAA	*spd_1267*
tuf_For	ATCACTGGTGCTGCTCAAA	*tuf*
tuf_Rev	CCTGACGTGAAAGAAGGATGT	*tuf*
**Probe**		
sodA	GCAAGCAAGGCTTCAAGGTCTTCACCGATTTCAGG	*sodA*
5s rRNA	GCGTTCTAGGGCTTAACTTCTGTGTTCGGCATGGG	*rrfA*

SUPPLEMENTAL FIGURES

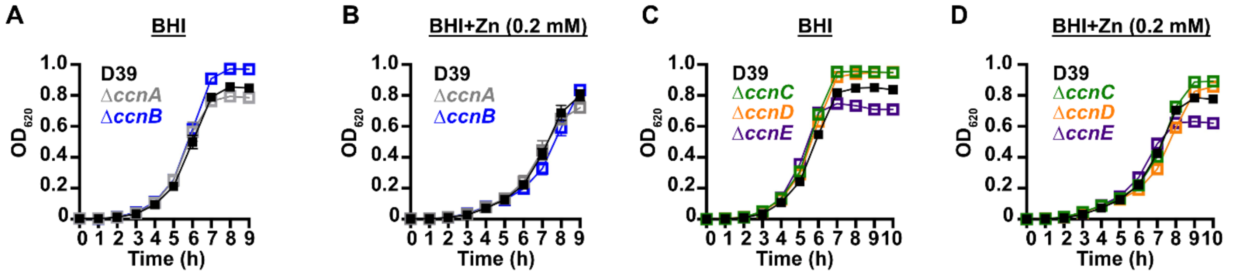

FIGURE S1. Growth phenotypes of *S. pneumoniae* D39 derived strains harboring deletion of individual *ccn* genes. Growth characteristics at 37°C under an atmosphere of 5% CO_2 in BHI broth alone (A,C) or with 0.2 mM $ZnSO_4$ (B, D) of the following strains: (A, B) IU781 (D39), NRD10073 (Δ*ccnA*), and NRD10074 (Δ*ccnB*); (C, D) IU781 (D39), NRD10075 (Δ*ccnC*), NRD10076 (Δ*ccnD*), and NRD10077 (Δ*ccnE*). Each point on the graph represents the mean OD_{620} value from three independent cultures. Error bars, which in some cases are too small to observe in the graph, represent the standard deviation (SD).

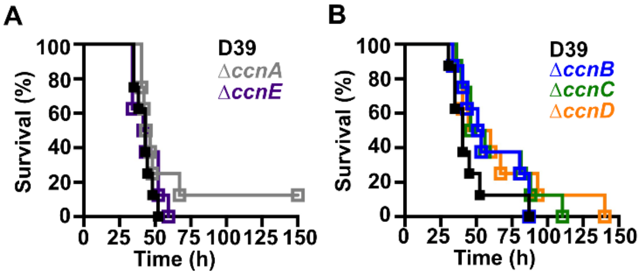

FIGURE S2. Virulence phenotypes of *S. pneumoniae* strains harboring deletion of individual *ccn* genes. Survival curve of ICR outbred mice after infection with ~10^7 CFU in a 50 μL inoculum of the following *S. pneumoniae* strains: (A) IU781 (D39), NRD10073 (Δ*ccnA*), and NRD10077 (Δ*ccnE*); (B) IU781 (D39), NRD10074 (Δ*ccnB*), NRD10075 (Δ*ccnC*), and NRD10076 (Δ*ccnD*). Eight mice were infected per strain. Disease progression of animals was monitored, the time at which animals reached a moribund state was recorded, and these mice were subsequently

euthanized as described in Materials and Methods. A survival curve was generated from this data and analyzed by Kaplan-Meier statistics and log rank test to determine P-values.

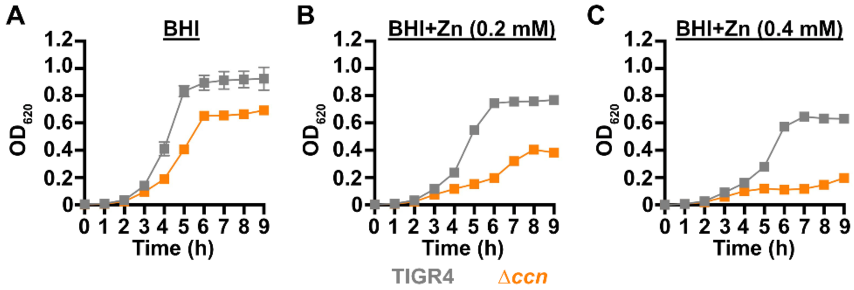

FIGURE S3. Growth phenotypes of *S. pneumoniae* TIGR4 derived strains harboring deletion of the *ccn* genes. Growth characteristics at 37°C under an atmosphere of 5% CO_2 in BHI broth alone (A) or with 0.2 mM (B) or 0.4 mM (C) $ZnSO_4$ of NRD10311 (TIGR4; TIGR4 *rpsL*$^+$-*rpsG*$^+$-*cat*) and NRD10346 (Δ*ccn*; TIGR4 *rpsL*$^+$-*rpsG*$^+$-*cat* Δ*ccnABCDE*). Each point on the graph represents the mean OD_{620} value from three independent cultures. Error bars, which in some cases are too small to observe in the graph, represent the standard deviation (SD).

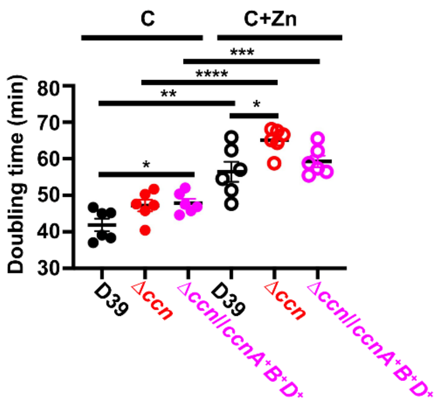

FIGURE S4. Doubling times of *S. pneumoniae* D39, Δ*ccnABCDE* mutant, or Δ*ccnABCDE* mutant strain complemented with *ccnA, ccnB,* and *ccnC* in C medium alone or supplemented with Zn. Shown are the mean doubling times during exponential growth of IU1781 (D39), NRD10176 (Δ*ccn)*, and NRD10396 (Δ*ccn//ccnA*$^{+}$*B*$^{+}$*D*$^{+}$) grown in C medium alone or supplemented with 0.2 mM ZnS04 as described in *Materials and Methods*. Doubling times for individual replicates are shown with solid lines indicating the mean of six different biological replicates and error bars denoting standard error of the mean (SEM). Statistical significance as determined by a Mann-Whitney test is indicate as * (P < 0.05), ** (P < 0.005), *** (P < 0.0005), or **** (P < 0.00005).

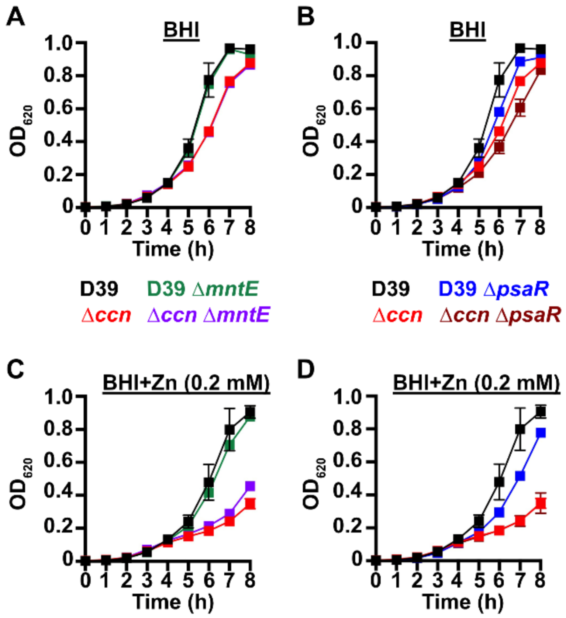

FIGURE S5. Growth phenotypes of *S. pneumoniae* D39 and derived strains harboring deletion of the *ccn* genes and/or *psaR* or *mntE*. Growth characteristics at 37°C under an atmosphere of 5% CO_2 in BHI broth alone (A, B) or with 0.2 mM $ZnSO_4$ (C, D) of the following strains: (A, C) IU781 (D39), NRD10176 (Δ*ccnABCDE*), NRD10448 (Δ*mntE*), and NRD10450 (Δ*ccnABCDE* Δ*mntE*); (B, D) IU781 (D39), NRD10176 (Δ*ccnABCDE*), NRD10447 (Δ*psaR*), and NRD10450 (Δ*ccnABCDE* Δ*psaR*). Each point on the graph represents the mean OD_{620} value from three independent cultures. Error bars, which in some cases are too small to observe in the graph, represent the standard deviation (SD).

SUPPLEMENTAL REFERENCES

1. D. Sinha, J. P. Frick, K. Clemons, M. E. Winkler, N. R. De Lay, Pivotal Roles for Ribonucleases in *Streptococcus pneumoniae* Pathogenesis. *mBio*, e0238521 (2021).
2. S. Ramos-Montanez *et al.*, Polymorphism and regulation of the *spxB* (pyruvate oxidase) virulence factor gene by a CBS-HotDog domain protein (SpxR) in serotype 2 *Streptococcus pneumoniae*. *Molecular Microbiology* **67**, 729-746 (2008).
3. L. T. Sham, K. R. Jensen, K. E. Bruce, M. E. Winkler, Involvement of FtsE ATPase and FtsX extracellular loops 1 and 2 in FtsEX-PcsB complex function in cell division of *Streptococcus pneumoniae* D39. *mBio* **4**, (2013).
4. D. Sinha *et al.*, S1 Domain RNA-Binding Protein CvfD Is a New Posttranscriptional Regulator That Mediates Cold Sensitivity, Phosphate Transport, and Virulence in *Streptococcus pneumoniae* D39. *Journal of Bacteriology* **202**, (2020).

5. M. A. Zafar, M. Kono, Y. Wang, T. Zangari, J. N. Weiser, Infant Mouse Model for the Study of Shedding and Transmission during *Streptococcus pneumoniae* Monoinfection. *Infection and Immunity* **84**, 2714-2722 (2016).
6. H. Tettelin *et al.*, Complete genome sequence of a virulent isolate of *Streptococcus pneumoniae*. *Science* **293**, 498-506 (2001).
7. K. E. Bruce, B. E. Rued, H. T. Tsui, M. E. Winkler, The Opp (AmiACDEF) Oligopeptide Transporter Mediates Resistance of Serotype 2 *Streptococcus pneumoniae* D39 to Killing by Chemokine CXCL10 and Other Antimicrobial Peptides. *Journal of Bacteriology* **200**, (2018).
8. H. C. Tsui *et al.*, Identification and characterization of noncoding small RNAs in *Streptococcus pneumoniae* serotype 2 strain D39. *Journal of Bacteriology* **192**, 264-279 (2010).
